# miRNA Expression Profiles in Ovarian Endometriosis and Two Types of Ovarian Cancer—Endometriosis-Associated Ovarian Cancer and High-Grade Ovarian Cancer

**DOI:** 10.3390/ijms242417470

**Published:** 2023-12-14

**Authors:** Maria Szubert, Anna Nowak-Glück, Daria Domańska-Senderowska, Bożena Szymańska, Piotr Sowa, Aleksander Rycerz, Jacek R. Wilczyński

**Affiliations:** 1 Department of Surgical and Oncologic Gynaecology, 1st Department of Gynaecology and Obstetrics, M. Pirogow’s Teaching Hospital, Medical University of Lodz, Wilenska 37 St., 94-029 Lodz, Poland; annanowak27@wp.pl (A.N.-G.); aleksander.rycerz@stud.umed.lodz.pl (A.R.); jrwil@post.pl (J.R.W.); 2 Club 35. Polish Society of Gynaecologists and Obstetricians, ul. Cybernetyki 7F/87, 02-677 Warsaw, Poland; 3 Department of Molecular Medicine, Medical University of Lodz, Pomorska 251 St., 92-213 Lodz, Poland; daria.domanska@umed.lodz.pl; 4 Research Laboratory CoreLab, Medical University of Lodz, Mazowiecka 6/8 St., 92-215 Lodz, Poland; bozena.szymanska@umed.lodz.pl; 5 Department of Pathology, M. Pirogow’s Teaching Hospital, Wilenska 37 St., 94-029 Lodz, Poland; noctee@wp.pl; 6 Department of Biostatistics and Translational Medicine, Medical University of Lodz, Mazowiecka 15 St., 92-215 Lodz, Poland

**Keywords:** miRNA, endometriosis, endometrial cyst, ovarian cancer, carcinogenesis

## Abstract

Endometriosis-associated ovarian cancer (EOC) consisting of endometrioid cancer and clear-cell ovarian cancer could be promoted by many factors. miRNAs, which are small, non-coding molecules of RNA, are among them. The aim of this study was to detect miRNAs connected with the malignant transformation of endometriosis. FFPE (formalin-fixed, paraffin-embedded) samples of 135 patients operated on for endometriosis and different types of ovarian cancer (EOC and HGSOC—high-grade serous ovarian cancer) were studied. Healthy ovarian tissue was used as a control group. From the expression panel of 754 miRNAs, 7 were chosen for further tests according to their ROC (receiver operating characteristic) curves: miR-1-3p, miR-125b-1-3p, miR-31-3p, miR-200b-3p, miR-502-5p, miR-503-5p and miR-548d-5p. Furthermore, other potentially important clinical data were analysed, which included age, BMI, Ca-125 concentration, miscarriages and deliveries and concomitant diseases such as hypertension, type 2 diabetes and smoking. Among the miRNAs, miR200b-3p had the lowest expression in neoplastic tissues. miR31-3p had the highest expression in women without any lesions in the ovaries. miR-502-5p and miR-548-5p did not differ between the studied groups. The examined miRNA panel generally distinguished significantly normal ovarian tissue and endometriosis, normal ovarian tissue and cancer, and endometriosis and cancer. The malignant transformation of endometriosis is dependent on different factors. miRNA changes are among them. The studied miRNA panel described well the differences between endometriosis and EOC but had no potential to differentiate types of ovarian cancer according to their origin. Therefore, examination of a broader miRNA panel is needed and might prove itself advantageous in clinical practice.

## 1. Introduction

Endometriosis is one of the most common gynaecological problems among women of reproductive age. Although benign in nature, it can seriously impact a woman’s life [1,2,3,4]. It has recently been proposed that endometriosis may behave like cancer, Furthermore, it may lead to reproductive tract cancers, especially ovarian cancer [5]. According to the literature, the ovarian form of endometriosis is most likely to progress into cancer, and this progression can take only 60–120 months in some cases [6].

Since the role of miRNA in the transition process from endometriosis into EOC has not been fully elucidated, we designed this study to test its role in both entities. In this research, we compared miRNA expression in different endometriosis tissues and endometriosis associated with ovarian cancer as well as in high-grade ovarian cancer (HGSOC). This is the first report of miRNA expression comparing these two entities with reference to high-grade serous ovarian cancer and healthy ovarian tissue.

EOC (endometriosis-associated ovarian cancer) consists of two histopathological subtypes: endometrioid and clear-cell ovarian cancer. EOC and endometriosis share some molecular features, such as different mutations, for example, in ARID1A, PTEN, KRAS and PIK3CA. There is also a well-described microsatellite instability and p53 loss in both entities [7,8,9]. The risk of development of EOCs accounts for 0.3–1.0% of women with diagnosed endometriosis [10]. Several other malignancies may arise from endometriosis foci mainly described in case reports, like mucinous tumours or carcinosarcoma [11,12]. Published case reports prove that ovarian endometriosis and other forms of the disease can develop into EOC [13,14]. This background leads to the conclusion that there is an urgent need for more sensitive and precise diagnostic methods of atypical forms of endometriosis and methods that can discriminate the susceptibility for EOC. miRNAs can serve in all these steps, especially when biological studies are combined with advanced statistical methods [15]. Every oncogenic transformation can be regulated by small non-coding microRNAs—molecules that influence gene expression (by up- or downregulation) on the post-transcriptional level [16]. Every miRNA may act on one or more mRNA (messenger RNA) transcripts and vice versa. Several miRNAs can regulate one mRNA, and its pleiotropic function has already been described in many cellular processes [17,18,19,20,21].

Data from our study broadened the basic knowledge of understanding endometriosis and the relationships between miRNAs and carcinogenesis. We hypothesised that miRNA expression could differ between endometriosis and ovarian cancer forms. To properly read its significance, we compared the expression of the chosen miRNA to healthy ovarian tissue and high-grade ovarian cancer.

## 2. Results

### 2.1. Patients’ Clinical and Histopathological Characteristics

Patients with EOC consisted of 26 patients with a mean age of 57 years. In the comparative group, HGSOC consisted of 29 patients with a mean age of 64.6 years; healthy patients were aged 56 years (n = 33). There were no differences in age between those groups, but there was a significant difference comparing the age of endometriosis patients, which is obvious taking into account endometriosis prevalence (Table 1).

Other factors like BMI, deliveries, miscarriages, comorbidities and nicotine abuse did not differ between groups. The staging of the ovarian cancer group according to the FIGO classification is shown in Table 2. In general, the FIGO staging comprises four stages. Stage I consists of a tumour limited to the ovaries or fallopian tubes; in stage II, tumour pelvic extension is present; and stage III and IV are called advanced (in stage III, abdomen or lymph node spread is seen; in stage IV, distant metastases are diagnosed) [22].

### 2.2. miRNA Expression

**Figure 1:**
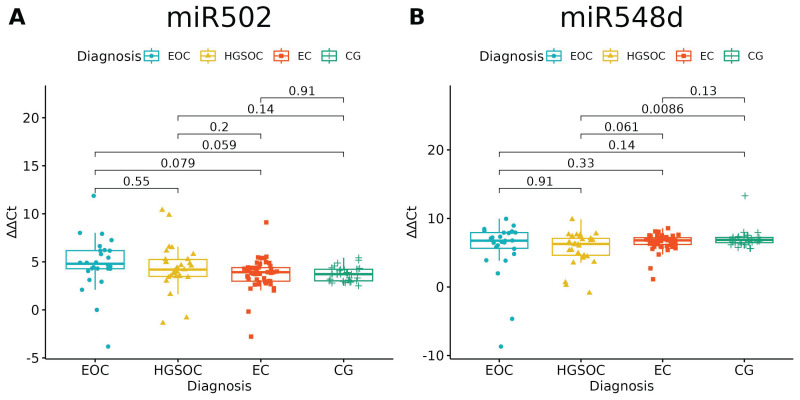
miR-502-5p (**A**) and miR-548-5p (**B**) expression in studied groups (ANOVA test). *p*-values between each group written above the box-plots. *Y*-axis shows Relative Quantification (RQ) value (_Δ Δ_ CT) to reference gene miR-191-5p. EOC—endometriosis-associated ovarian cancer; HGSOC—high-grade ovarian cancer; EC—endometrial cyst; CG—control group. The remaining microRNAs showed differential expression between groups (Table 3), which we further explain in Figure 2, Figure 3 and Figure 4.

A total of 7 miRNAs with altered expression profiles were chosen out of 754 human miRNA genes. hsa-miR-191-5p (assay ID: 477952_mir) served as a reference gene (calibrator) which was chosen according to the literature: https://mirbase.org/cgi-bin/mirna_entry.pl?acc=MI0000465 (URL accessed on 19 August 2019) [23,24,25]. A screening cohort was created of randomly chosen FFPE ovarian endometriosis samples (OE) (n = 20) and controls (CG) (n = 20) and of the ovarian cancer group (n = 20 + 20) to conduct the first miRNA expression reaction, with 754 miRNAs provided by the manufacturer (Supplementary Table S1; as mentioned above, the raw data are available also under https://doi.org/10.3390/ijms23094660 (accessed on 1 February 2023) and are stored in the repository under ijms-23-04660-s001.zip (532K), GUID: 8CEB1874-B810-4EF0-AC25-31E7787CCA87). MiRNAs with altered expression profiles (RQ = Relative Quantification compared to reference hsa-miR-191-5p and normalised versus control group) were chosen according to analysis of their PCR amplification curves and analysis of the literature backgrounds. An altered expression profile meant at least a 1.5-fold change in the expression level, with visible differences between groups. In addition, miR-1-3p with only a 1.4-fold change was included in the second step of analysis because of its role described in endometriosis (studies reporting its upregulation in endometriosis). Chosen miRNAs were as follows-Table 3. (detailed description in Section 2):

Of the investigated microRNAs, only miR-502-5p and miR-548d-5p had no significantly different expressions between the studied groups (*p* = 0.0697 and *p* = 0.0601, respectively). There is a difference between HGOC and CG in miR-548d-5p Relative Quantification, but one should bear in mind that healthy ovarian tissue and high-grade ovarian cancer tissue served as comparators for endometriosis and endometriosis-associated ovarian cancer (Figure 1, Table 4).

**Figure 2:**
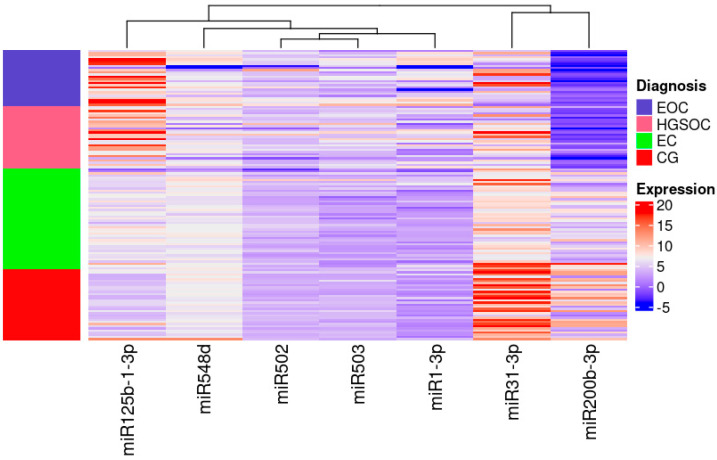
Heatmap of the Relative Quantification of investigated miRNAs.

miR-31-3p had the highest expression in women without any lesions in ovaries and miR-200b-3p had the lowest expression in women with ovarian cancer irrespective of its pathological origin (Figure 2).

High expression is indicated in red, low expression is indicated in blue and the left column presents the areas matching each group on the heat map. Colours are defined on the right side of the figure.

**Figure 3:**
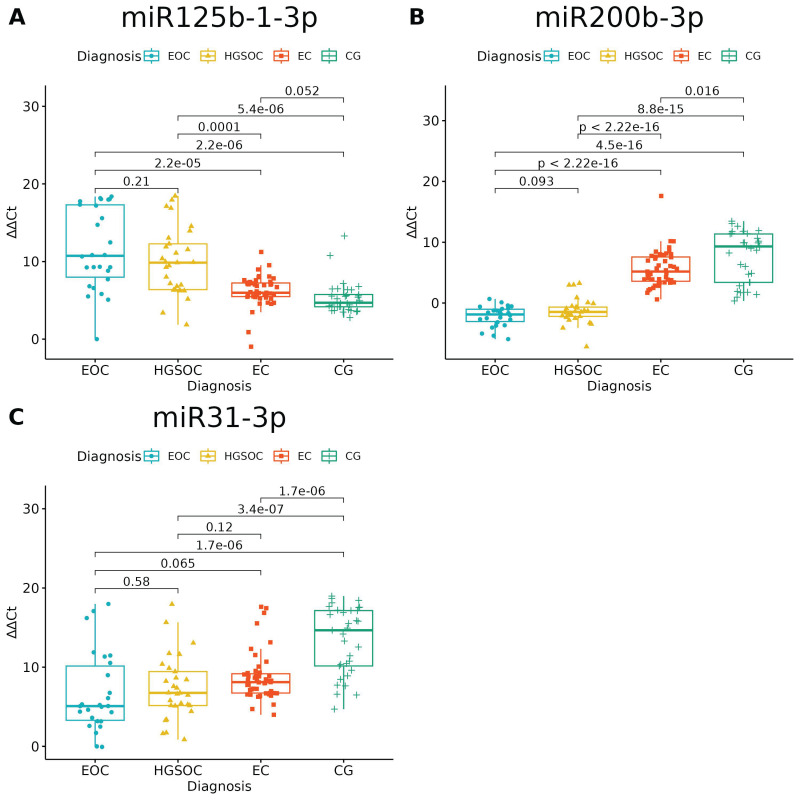
miRNAs with significantly altered expression between endometriosis-associated ovarian cancer (EOC) and ovarian endometriosis (EC) (ANOVA test with post hoc *t*-test). *p*-Values between each group written above the box-plots. *Y*-axis shows Relative Quantification (RQ) value (_Δ Δ_ CT) to reference gene miR-191-5p. EOC—endometriosis-associated ovarian cancer, HGSOC—high-grade ovarian cancer, EC—endometrial cyst, CG—control group; (**A**)–miR-125-b-1-3p; (**B**)–miR-200b-3p; (**C**)–miR-31-3p.

**Figure 4:**
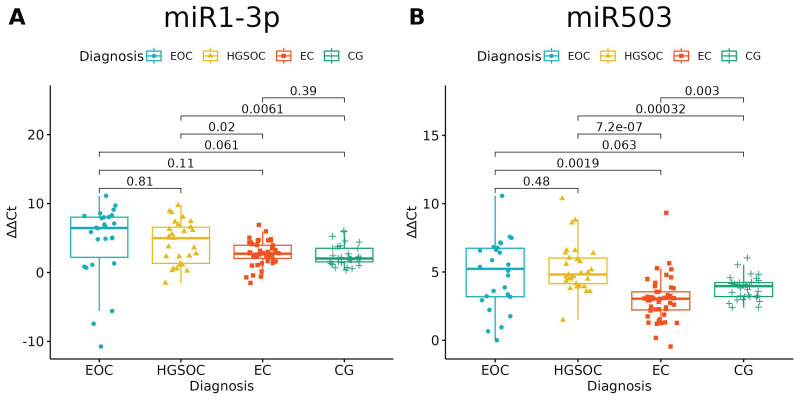
(**A**) miR-1-3p significantly distinguished only benign and malignant tissue; miR-503-5p (**B**) had a lower expression in ovarian endometriosis than in all types of cancer (ANOVA test with post hoc *t*-test). *p*-Values between each group written above the box-plots. *Y*-axis shows Relative Quantification (RQ) value (_Δ Δ_ CT) to reference gene miR-191-5p. EOC—endometriosis-associated ovarian cancer, HGSOC—high-grade ovarian cancer, EC—endometrial cyst, CG—control group.

None of the studied miRNAs discriminated well the EOC from HGSOC (Figure 3 and Figure 4), but miR-125b-1-3p, miR-200b-3p and miR-31-3p had significant differences in expression between ovarian endometriosis and EOC (Figure 3). In ovarian endometriosis, miR-125b-1-3p and miR-503-5p had lower expressions than in cancer, miR-200b-3p had a higher expression, and miR-1-3p had a comparable expression in benign tissues (ovarian endometriosis and healthy ovarian tissue, with a higher expression in cancers) (Figure 4).

Changes in miR-200b-3p and miR-31-3p could well describe carcinogenesis—the highest expression was in healthy tissue, the next-highest expression was in the benign tissue and the lowest expression was in cancer tissue. The opposite pattern is seen for miR-125b-1-3p: the highest expression was in cancer and the lowest expression was in healthy tissue.

#### The miRNA Enrichment Analysis and Annotation Tool

Since the knowledge of miRNAs and their interactions is rapidly growing, there are several databases and analytical tools that enable the comparison of sets of miRNAs. One of them is miEAA. The miRNA Enrichment Analysis and Annotation Tool (miEAA) facilitates the functional analysis of sets of miRNAs. It is based on GeneTrail (https://genetrail.bioinf.uni-sb.de/) (accessed on 1 October 2023). GeneTrail is a large platform to compare genex expression data, miRNA data and proteins data and to process functional analyses and provide possible connections between molecules. In contrast to GeneTrail, miEAA is tailored for miRNA precursors and mature miRNAs of multiple frequently investigated species. Creating functional analysis, one should remember to correctly input species [26]. MiEAA has multiple predefined categories selected from numerous databases and publications. There are several *p*-value adjustments available, the default of which is the FDR (Benjamini–Hochberg) adjustment.

**Figure 5:**
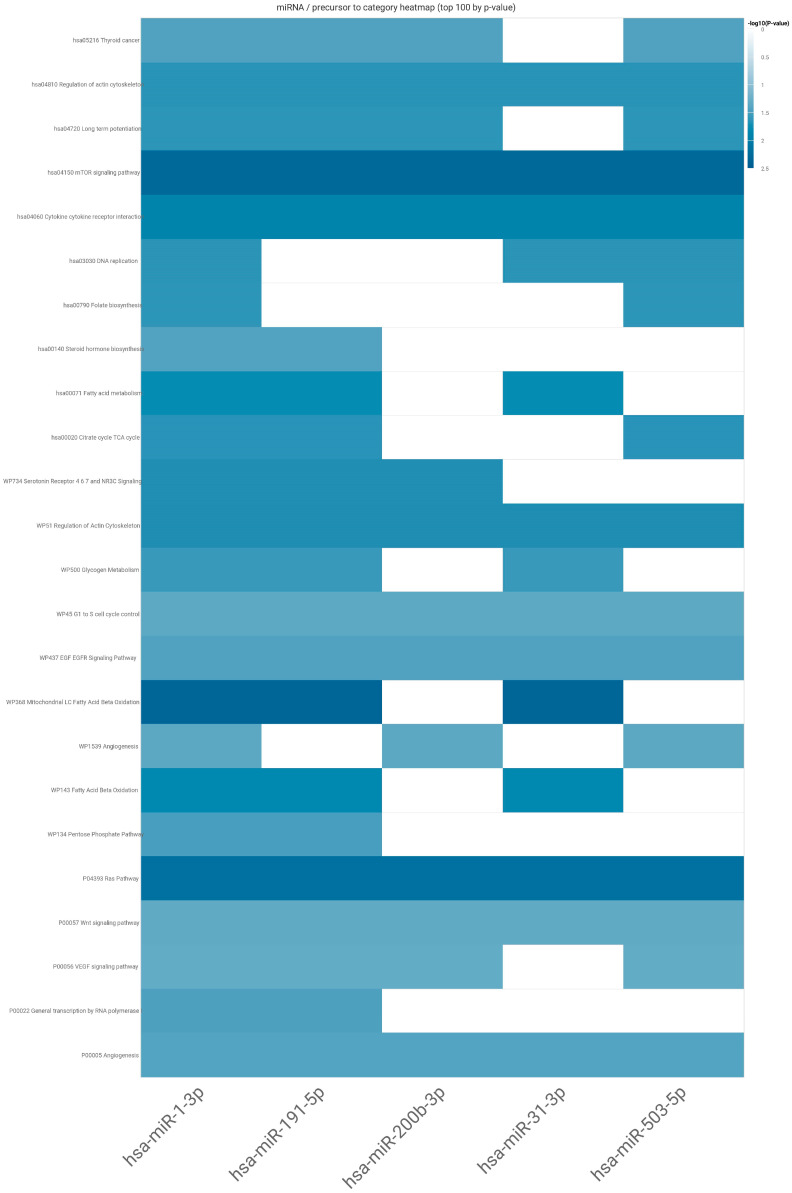
The heatmap of connections between studied miRNAs and the most significant pathways (rows—subcategories of possible pathways, displayed in log base; columns—the studied miRNAs).

Firstly, we selected the miRNA for analysis and the type of enrichment analysis, and then we put our set of studied miRNA into webtool—miEAA version 2.1. The results from the analysis generated as an pdf and excel file can be found in Supplementary miEAA Analysis Results (PDF file) and Table S2. In the Supplementary Materials, we also provide analysis of potential targets for each individual studied miRNA (Supplementary Table S3). Here, we present a heat map according to which the set of studied miRNAs could impact mTOR and RAS signalling pathways (Figure 5).

## 3. Discussion

Our study is one of the first to test miRNAs in different forms of endometriosis (see [27]) and ovarian cancer samples divided according to their origin. The thesis on the malignant transformation of endometriosis is broadly studied, but for the first time, the same panel of miRNAs was examined in each form of this disease and different forms of ovarian cancer. This study’s novelty relies on the use of healthy ovarian tissue as a control group and high-grade ovarian cancer as a comparative group. It is suggested that endometriosis is responsible for the increase in ovarian cancer risk by up to 3.4 times for clear-cell cancer, especially the ovarian form of the disease [14]. The exact risk is still unknown; the data are heterogeneous and depend on the nature of the study (prospective, retrospective, analysis of national cohorts). Three of the studied miRNAs, miR-200b-3p, miR-31-3p and miR-125b-1-3p, were well differentiated between healthy tissue, ovarian endometriosis which is a benign disease and ovarian cancer that could originate from endometriosis. However, we failed to distinguish EOC from HGSOC by comparing miRNA expression solely.

It is well known that one miRNA might target several mRNAs (messenger RNAs) acting as its activator or inhibitor. The regulation of gene expression is very likely mediated by miRNA networks rather than individual miRNAs [28]. The miRNAs tested in our study proved through enrichment functional analysis to act especially on mTOR and RAS pathways. As other authors have stated, the functional interaction between miRNAs might be defined by the number of their common targets [29]. The analysis conducted with the use of miEAA revealed almost 26 potential signalling pathways for our set of miRNAs.

Most ovarian neoplasm cases are of epithelial origin, with five subtypes: high-grade serous ovarian cancer, low-grade serous cancer, mucinous cancer, endometrioid cancer and clear-cell cancer. Only two of them are identifiable as originating from endometriosis [30]. One of the most important driver events in ovarian cancer associated with endometriosis is the *ARID1A* loss-of-function mutation (responsible for chromatin remodelling). It can be inhibited by miR-31-3p, as proven by Lu et al. in a study on head and neck cancer [31]. On the contrary, in high-grade serous ovarian cancer, a high frequency of the *TP53* mutation dominates. There is also the homologous recombination deficiency (HRD), including *BRCA1* and *BRCA2* mutations found in up to 20% of affected women [32]. In our study, miR-31-3p had the lowest expression in EOC, according to the results published by Nagaraja et al. [33] who had found miR-31-3p deregulated in clear-cell cancer compared to the normal ovarian surface epithelium. In one of the first papers on miRNAs’ role in cancer, Lu et al. stated that miRNA profiles can reflect the developmental lineage and differentiation state of the tumours [34]. Our results confirm this thesis that miRNA can act differently in tissues of non-clonal origin.

The heatmap generated through miEAA revealed that the studied set of miRNAs acts on both very well-known tumourigenesis pathways. The RAS/RAF/MEK/ERK (MAPK) pathway plays a role in ovarian carcinogenesis. It is probably the second step (after the first: mutations in ARID1A) leading from benign tissue to uncontrolled proliferation. The next one, very well described in carcinogenesis—the PI3K/AKT/mTOR pathway—is one of the commonly described pathways interfering with the MAPK pathway, with multiple levels of mutual interactions [35]. miRNAs are among them. The aberrant activation of RAS leads to the uncontrolled induction of several downstream signalling pathways such as RAF-1/MAPK (mitogen-activated protein kinase), PI3K phosphoinositide-3 kinase (PI3K)/AKT and many others involved in cell proliferation. As a result of its action, tumorigenesis and cancer cell propagation start [36].

After years of studies, endometriosis has also been described with its own miRNA signature [37]. Bendifallah et al. screened saliva samples from endometriosis patients using the NGS method (next-generation sequencing) and proved that almost 100 miRNAs are up- or downregulated in endometriosis patients [37]. Saliva as a medium was used before, but only for nasopharyngeal cancers. miRNAs in saliva were downregulated in cancers, which directly impacted the oncogene TP53 [38]. The test with twelve dysregulated miRNAs had almost 100% sensitivity in detecting nasopharyngeal carcinoma [38]. The study of Wu et al. also supported the thesis that there is a general downregulation of miRNAs in tumours compared with normal tissues [34,38]. Our results partially follow this hypothesis: miR-200b-3p and miR-31-3p were downregulated in ovarian cancers, but none of the studied miRNAs could distinguish between EOC and HGSOC. Hence, HGSOC tissue was included in this study to prove that there could be a possible dysregulation in miRNA expression leading from normal tissue through endometriosis to EOC but not to HGSOC; we failed to prove this hypothesis.

The epithelial–mesenchymal transition (EMT) is a process of changes in signalling pathways that determine biological processes to allow cells to gain mesenchymal phenotypes [39]. The EMT was found to be disturbed in endometriosis and it could be responsible for acquiring malignant features by tissues [40]. It has been proven that the *ARID1-A* mutation influences this physiological process in the ectopic endometrium, leading to amplification of the EMT. The forced expression of miR-1 or miR-200 inhibited both EMT and tumourigenesis in the in vitro study [39]. One of the miRNA families that suppress cancer is the miR-200 family. miR-200 acts on the epithelial–mesenchymal transition, causing its inhibition [41]. Decreased miR-200 expression was associated with the acquisition of cancer stem cells and tumour-initiating capacity, as proven on breast cancer cell lines by Shimono et al. [42]. In our study, mir-200b was also downregulated in ovarian cancer, and its decreasing expression was visible in endometrial cysts compared to the healthy ovarian tissue.

However, one should remember that the family of human miRNA-200 contains five members, miR-200a, miR-200b, miR-200c, miR-141 and miR-429, that can act differently on the same level. They are divided into two clusters. MiR-200b, miR-200a and miR-429 (cluster I) are located on chromosome 1, and their function seems comparable within the cluster [43]. As a whole family, miRNA-200 can promote or inhibit genes responsible for cell growth and cell apoptosis. They were proposed to serve as a promising therapeutic target [44]. Ibrahim et al. especially focused on miR-200c and proved that it can influence cell proliferation and colony formation—two steps necessary for metastasis in serous ovarian cancer [44]. In another study, the upregulation of miR-200b and miR-200c promoted cancer cell death in the presence of cisplatin [45]. Cisplatin- or carboplatin-based chemotherapy is the most important step in the first-line treatment of ovarian cancer. Significantly, miR-200b and miR-200c reversed cisplatin resistance by targeting DNA methyltransferases [45].

In light of our findings and the already mentioned results, the following hypothesis could be proposed: the low expression of miR200b could influence prognosis and survival through chemoresistance and the ability to form metastases.

Another miRNA well recognised in cancer studies is miR-125. The upregulation of miR-125b-5p significantly decreased tumour growth in combination with cisplatin in a mouse model [45,46]. This fact could be useful in future therapy in all squamous epithelial cancers in which miR-125b-5p-based therapy is the first choice. miR-125b-5p acts mainly as a tumour suppressor in many different ways, for example, interacting with VEGF (vascular endothelial factor), PARP 1 (poly-ADP-ribose-polymerase 1) and PRXL2A (peroxiredoxin like 2A gene, responsible for anti-oxidative processes) [47]. In our study, we found a significant difference between its expression in endometriosis and EOC, which confirms its role in carcinogenesis. It is worth mentioning that miR-503-5p was also significantly upregulated in EOC and HGOC in our group as compared with healthy tissue, but in endometriosis, its expression was much lower than in the healthy ovary. It has already been described as being decreased in endometriosis through hypermethylation [48]. This contributes to proliferation, resistance to apoptosis, extracellular matrix (ECM) contractility and angiogenesis [48]. miR-503-5p suppression plays a critical role in CD97 expression (a member of the epidermal growth factor (EGF)—seven-transmembrane family, expressed in many cancers) and the related JAK2/STAT3 pathway for enhancing the metastasis of paclitaxel-resistant ovarian cancer cells [49].

There are a lot of classification systems of endometriosis (rASRM, EFI, #ENZIAN) but all those classifications have several well-recognisable disadvantages. The symptoms do not correlate with the advancement of the disease, factors leading to progression of the disease or worsening of the symptoms are not well described, and carcinogenesis from endometrial changes through atypical endometriosis into EOC is not captured in any classification system [50]. That is why it is difficult to indicate which patient will benefit from radical surgery or where small endometrial cysts could be left without surgical treatment. Studies like ours help to gain knowledge in this field.

We suggest that the trends in the changes in miRNAs in endometriosis are similar to EOC, and the downregulation of miR-200b-3p, miRNA31-3p and miRNA125b-1-3p should be further studied in patients with confirmed carcinogenesis in endometriosis foci.

To date, Suryawanshi et al. proved differences in the miRNA signature between endometriosis and EOC and HGSOC in plasma samples [51], and Braicu et al. proved differences in endometriosis and EOC formalin-fixed paraffin-embedded samples [15], but none of the mentioned researchers tested patients with proven anamnesis of carcinogenesis in endometriosis foci. This is also a limitation of our study. We did not test samples of ovarian cancer developed in the same patient, in whom endometriosis had previously been diagnosed and FFPE endometriosis samples were possible to obtain. To obtain this kind of sample, one should plan the study over at least a dozen years. Secondly, the number of clear-cell cancer samples was relatively low in our group of EOC. This burden could be omitted by planning large, multicentre studies or studies in specific cohorts of patients [52].

## 4. Materials and Methods

This study’s methods are described in detail and published under https://doi.org/10.3390/ijms23094660 (accessed on 1 February 2023). All raw data are available and stored in the repository under ijms-23-04660-s001.zip (532K), GUID: 8CEB1874-B810-4EF0-AC25-31E7787CCA87. Below, we describe the key steps of this study.

Data for this analysis were obtained from 135 patients hospitalised and diagnosed with ovarian endometriosis and ovarian cancer between 2010 and 2018 at the Department of Surgical and Oncologic Gynaecology, Medical University of Lodz, Poland, for whom histopathological FFPE blocks (formalin-fixed, paraffin-embedded) were identified. EOC endometrioid ovarian cancer samples and clear-cell ovarian cancer were classified. Reassessment by a pathologist was conducted to confirm the diagnosis and to indicate the right place to obtain the tissue from FFPE blocks. The following data were chosen for statistical analysis to ensure proper accordance with inclusion and exclusion criteria: age, BMI, menopausal status, menarche, pregnancy and delivery status, comorbidities, addictions, family history, data about hormonal therapies, data about other cancers and previous chemotherapy, FIGO classification for ovarian cancer.

Exclusion criteria were as follows: known hormonal therapy before surgery (endometriosis patient should be at least 3 months without hormonal treatment), known diagnosis of synchronous cancer, chemo- or radiotherapy in the past. The study group consisted of 26 patients with endometriosis-associated ovarian cancer = EOC (21 endometrial ovarian cancer, 5 clear-cell cancer), 29 patients with high-grade serous ovarian cancer = HGSOC, 47 patients with ovarian endometrial cyst (EC), with 33 patients with “healthy ovarian tissue” in the control group = CG (ovaries removed from menopausal women along with uterus due to benign conditions, such as large myomas, abnormal uterine bleeding that causes indication for surgery after menopause). This study was approved by the Bioethics Committee of the MU of Lodz (Ref. No. RNN 403/18/KE).

### 4.1. Preparation of Tissue Samples

Affected areas in FFPE blocs were precisely identified by an experienced pathologist, and then they were carefully dissected on a semiautomatic microtome in a way that minimises the risk of contaminating them with nonaffected tissue. Normal ovarian tissue harvested after TLH (total laparoscopic hysterectomy) or AH (abdominal hysterectomy) for benign diseases other than endometriosis or adenomyosis, mostly for myomas, formed the control group. The decision to use ovarian tissue as the control group resulted from the fact that miRNA expression is tissue-specific (https://ccb-web.cs.uni-saarland.de/tissueatlas) (accessed on 19 August 2019) [53].

### 4.2. RNA Extraction from FFPE Samples

Isolation of total RNA from one or two 10–20 μm thick tissue slices was performed using the High Pure FFPE Isolation Kit (Roche, Germany). The tissue was deparaffinised with 100% xylene, and then washed with 100% ethanol and dried at 55 °C for 10 min. Protease digestion was performed at 55 °C, overnight. The tissue was purified using the process recommended by the manufacturer: 325 µL of Binding Buffer and 325 µL of Binding Enhancer were added, the mixture was fed into the columns and washed twice with Wash Buffer, and then it was eluted with 50 µL of Elution Buffer. Determinations of the RNA product yield and quality were performed using a PicoDrop spectrophotometer (PicoDrop Limited, Hinxton, UK). The purified total RNA was either immediately used in the process of cDNA synthesis or stored at −80 °C until use.

### 4.3. Determinations of the miRNA Gene Expression Profile

First, the expression of miRNA was screened using TaqMan^®^ Human MicroRNA Array A and B (Applied Biosystems, Foster City, CA, USA). Megaplex™RT Primers Human Pool A and B, purchased from Applied Biosystems, were used to prepare the reaction of reverse transcription as specified in the manufacturer’s instructions. The 7900HT Fast Real-Time PCR System (Applied Biosystems, CA, USA) was used for amplifications conducted under the following conditions: 2 min at 50 °C, 10 min at 94.5 °C and then 40 cycles of 30 s at 97 °C and 1 min at 57 °C each. In the first step, the levels of expression were assessed for 754 human miRNA genes in the screening cohort of randomly selected FFPE samples from ovarian endometriosis (OE) patients (n = 20) and controls (CG) (n = 20) and in samples of ovarian cancer (n = 20 for high-grade serous ovarian cancer + 20 for endometrioid ovarian cancer). miRNAs with altered expression profiles (on the basis of 1.5-fold change and PCR amplification curves, and confirmed in available reports) were chosen for further investigations.

### 4.4. RT-qPCR with Individual cDNA

An individual, quantitative RT-qPCR study was conducted using the TaqMan Advanced miRNA Reverse Transcription Kit (Thermo Fisher, Karlsruhe, Germany), as specified in the protocol provided by the manufacturer. Briefly, 10 ng of total RNA was used in poly(A) tailing reaction, followed by adapter ligation and reverse transcription. To uniformly increase the cDNA amount for all miRNAs (miR-Amp reaction), the diluted RT product was preamplified and was then either immediately used in the qPCR study or stored at −20 °C.

### 4.5. MicroRNA Assays

The selected miRNA was quantified using TaqMan Advanced MicroRNA Assays (Applied Biosystems), hsa-miR-1-3p (Assay ID: 477820_mir), hsa-miR-31-3p (Assay ID: 478012_mir), hsa-miR-125b-1-3p (Assay ID: 478665_mir), hsa-miR-200b-3p (Assay ID: 477963_mir), hsa-miR548d (Assay ID: 480870_mir), hsa-miR-502-5p (Assay ID: 47954_mir), hsa-miR-503-5p (Assay ID: 47143_mir) and hsa-miR-191-5p (Assay ID: 477952_mir), used as reference genes. An amount of 10 μL of qPCR reaction mixture contained 2.5 μL of diluted RT product, 5 μL of TaqMan Fast Advanced PCR Master Mix (Applied Biosystems) and 0.5 μL of TaqMan miRNA Assay (20×). The reactions were conducted using a 96-well plate, at 95 °C for 10 min, followed by 40 cycles at 95 °C for 5 s and at 60 °C for 20 s each, in duplicates. The relative miRNA quantification was analysed with comparative Ct. The calculated level of miRNA was 2−ΔΔC t, while the analysis of relative expression for the studied gene is shown as an n-fold change in gene expression normalised to a reference gene relative to the control.

### 4.6. miRNA Enrichment Analysis and Annotation Tool

To establish the role of selected miRNAs, we performed analysis in accordance with miRNA Enrichment Analysis and Annotation Tool (miEAA version 2.1)—https://ccb-compute2.cs.uni-saarland.de/mieaa/annotation/?jobid=d321af0e-ef52-4716-b17f-a20a241d3b64 (accessed on 1 October 2023). A heatmap was generated to visualise all the functional categories that are combined with least one of the miRNAs.

### 4.7. Statistical Analysis

Statistical analysis was performed using RStudio 4.3.0. *p*-Values < 0.05 were considered statistically significant. Shapiro–Wilk test was used to test normality. Differences between groups were tested using the ANOVA test and *t*-test. Benjamini and Hochberg’s method was used to adjust the *p*-value.

## 5. Conclusions

Our study should be considered as a preliminary report which shows potential targets for future research. The characterisation of miRNAs in EOC, HGSOC and simultaneously in endometriosis foci could shed light on the transition from healthy ovarian tissue through benign changes and atypical forms into ovarian cancer. We observed a profound downregulation of miRNA-200b and miRNA-31-3p in cancerogenic lesions compared to healthy ovarian tissue and endometriosis tissue. Therefore, it can be hypothesised that the loss of both key miRNAs may form the basis of carcinogenesis in endometriosis. Further investigation may clarify the role of other miRNAs, especially in predicting which patient with endometriosis has a higher risk of transformation into a malignant disease. This fact could give the basis for improvement in endometriosis therapy and could enable the decision of radical surgical treatment of endometriosis. Adding functional analyses to further studies would improve the understanding of miRNA interactions on different signalling pathways.

## Figures and Tables

**Table 1:** Population characteristics (deliveries, miscarriages presented as medians, hypertension, smoking presented as percentages, n—number of patients; ^1^ EC—endometrial cyst, ^2^ EOC—endometriosis-associated ovarian cancer, ^3^ HGSOC—high-grade ovarian cancer, ^4^ CG—control group; *p* value statistically important if <0.05—ANOVA test).

	^1^ EC n = 47	^2^ EOC n = 26	^3^ HGSOC n = 29	^4^ CG n = 33	*p*
Age (years)	38.92 ± 13.51	56.80 ± 12.46	64.31 ± 11.28	58.10 ± 12.52	<0.05
BMI (kg/m^2^)	25.21 ± 3.91	27.38 ± 4.25	28.70 ± 3.19	27.55 ± 4.05	>0.05
CA125 (U/mL)	63.52 ± 68.90	143.18 ± 118.81	884.47 ± 922.59	n/a	<0.05
Deliveries	0	1.5	2	2	<0.05
Miscarriages	0	0	0	0	>0.05
Hypertension	13%	20%	19%	18%	>0.05
Smoking	14%	16%	18%	18%	>0.05

**Table 2:** FIGO (The International Federation of Gynaecology and Obstetrics) staging in EOC and HGSOC, n—number of patients.

	Stage	I	II	III
Cancer				
EOC (n)		15	5	6
HGSOC (n)		4	9	16

**Table 3:** Names and accession numbers of studied miRNA.

Name in Database	Accession Number
hsa-miR-125b-1-3p	MIMAT0004592
hsa-miR-31-3p	MIMAT0004504
hsa-miR-1-3p	MIMAT0000123
hsa-miR-191-5p	MIMAT0000440
hsa-miR-200b-3p	MIMAT0000318
hsa-miR-502-5p	MIMAT0002873
hsa-miR-503-5p	MIMAT0002874
hsa-miR-548d-5p	MIMAT0004812

**Table 4:** Mean of the Relative Quantification of studied microRNAs (with results of ANOVA). ^1^ EC—endometrial cyst; ^2^ EOC—endometriosis-associated ovarian cancer; ^3^ HGSOC—high-grade ovarian cancer; ^4^ CG—control group. *p* value (ANOVA test) statistically significant if <0.05; RQ presented as values in relation to reference gene.

	^1^ EC	^2^ EOC	^3^ HGSOC	^4^ CG	*p* Value
miR1-3p	2.74	3.71	4.26	2.43	0.0106
miR125b-1-3p	6.22	11.16	9.95	5.31	<0.0001
miR200b-3p	5.60	−2.46	−1.31	7.69	<0.0001
miR31-3p	8.75	7.48	7.37	13.40	<0.0001
miR-502-5p	3.74	4.50	4.39	3.71	0.0697
miR-503-5p	2.98	4.42	5.26	3.82	<0.0001
miR-548d-5p	6.55	5.37	5.62	6.99	0.0601

## Data Availability

The data presented in this study are available in this article (and Supplementary Materials).

## Supplementary Materials

The supporting information can be downloaded at https://www.mdpi.com/article/10.3390/ijms242417470/s1.
