# Supplementary material for: miRNA Expression Profiles in Ovarian Endometriosis and Two Types of Ovarian Cancer—Endometriosis-Associated Ovarian Cancer and High-Grade Ovarian Cancer
Source: Int J Mol Sci. 2023 Dec 14;24(24):17470. doi: 10.3390/ijms242417470 (PMC10743418; doi:10.3390/ijms242417470)
Supplement: Supplementary file 1 [file ijms-24-17470-s001.zip › miEAA - miRNA Enrichment and Annotation -- Analysis results(1).pdf]

Category	Subcategory
Pathways (miRWalk)	WP368 Mitochondrial LC Fatty Acid Beta Oxidation
Pathways (miRWalk)	hsa04150 mTOR signaling pathway
Pathways (miRWalk)	P04393 Ras Pathway
Pathways (miRWalk)	hsa04060 Cytokine cytokine receptor interaction
Pathways (miRWalk)	WP143 Fatty Acid Beta Oxidation
Pathways (miRWalk)	hsa00071 Fatty acid metabolism
Pathways (miRWalk)	WP51 Regulation of Actin Cytoskeleton
Pathways (miRWalk)	WP734 Serotonin Receptor 4 6 7 and NR3C Signaling
Pathways (miRWalk)	hsa00020 Citrate cycle TCA cycle
Pathways (miRWalk)	hsa04810 Regulation of actin cytoskeleton
Pathways (miRWalk)	hsa03030 DNA replication
Pathways (miRWalk)	hsa00790 Folate biosynthesis
Pathways (miRWalk)	hsa04720 Long term potentiation
Pathways (miRWalk)	WP500 Glycogen Metabolism
Pathways (miRWalk)	WP134 Pentose Phosphate Pathway
Pathways (miRWalk)	P00022 General transcription by RNA polymerase I
Pathways (miRWalk)	WP437 EGF EGFR Signaling Pathway
Pathways (miRWalk)	hsa05216 Thyroid cancer
Pathways (miRWalk)	hsa00140 Steroid hormone biosynthesis
Pathways (miRWalk)	P00005 Angiogenesis
Pathways (miRWalk)	WP1539 Angiogenesis
Pathways (miRWalk)	WP45 G1 to S cell cycle control
Pathways (miRWalk)	P00057 Wnt signaling pathway
Pathways (miRWalk)	P00056 VEGF signaling pathway

miEAA - miRNA Enrichment and Annotation -- Analysis results

Enrichment	P-value	P-adjusted	Q-value	Expected	Observed
over-represented	0.0046009	0.0046009	0.580125	0.396117	3
over-represented	0.0053397	0.0053397	0.580125	1.56117	5
over-represented	0.0068138	0.0068138	0.580125	1.64272	5
over-represented	0.0131628	0.0131628	0.580125	1.88738	5
over-represented	0.0156319	0.0156319	0.580125	0.605825	3
over-represented	0.0173805	0.0173805	0.580125	0.629126	3
over-represented	0.0188974	0.0188974	0.580125	2.03883	5
over-represented	0.0192418	0.0192418	0.580125	0.652427	3
over-represented	0.0222486	0.0222486	0.580125	0.687379	3
over-represented	0.0226781	0.0226781	0.580125	2.12039	5
over-represented	0.023309	0.023309	0.580125	0.699029	3
over-represented	0.0235694	0.0235694	0.580125	0.256311	2
over-represented	0.0237104	0.0237104	0.580125	1.32816	4
over-represented	0.0266667	0.0266667	0.580125	0.733981	3
over-represented	0.0324646	0.0324646	0.580125	0.302913	2
over-represented	0.034876	0.034876	0.580125	0.314563	2
over-represented	0.0366408	0.0366408	0.580125	2.3534	5
over-represented	0.0369537	0.0369537	0.580125	1.50291	4
over-represented	0.0373596	0.0373596	0.580125	0.326214	2
over-represented	0.0383244	0.0383244	0.580125	2.3767	5
over-represented	0.0443531	0.0443531	0.580125	0.885437	3
over-represented	0.0456377	0.0456377	0.580125	2.4699	5
over-represented	0.0476165	0.0476165	0.580125	2.4932	5
over-represented	0.0493017	0.0493017	0.580125	1.63107	4

miRNAs/precursors

hsa-miR-31-3p; hsa-miR-1-3p; hsa-miR-191-5p
hsa-miR-31-3p; hsa-miR-1-3p; hsa-miR-191-5p; hsa-miR-200b-3p; hsa-miR-503-5p
hsa-miR-31-3p; hsa-miR-1-3p; hsa-miR-191-5p; hsa-miR-200b-3p; hsa-miR-503-5p
hsa-miR-31-3p; hsa-miR-1-3p; hsa-miR-191-5p; hsa-miR-200b-3p; hsa-miR-503-5p
hsa-miR-31-3p; hsa-miR-1-3p; hsa-miR-191-5p
hsa-miR-31-3p; hsa-miR-1-3p; hsa-miR-191-5p
hsa-miR-31-3p; hsa-miR-1-3p; hsa-miR-191-5p; hsa-miR-200b-3p; hsa-miR-503-5p
hsa-miR-1-3p; hsa-miR-191-5p; hsa-miR-200b-3p
hsa-miR-1-3p; hsa-miR-191-5p; hsa-miR-503-5p
hsa-miR-31-3p; hsa-miR-1-3p; hsa-miR-191-5p; hsa-miR-200b-3p; hsa-miR-503-5p
hsa-miR-31-3p; hsa-miR-1-3p; hsa-miR-503-5p
hsa-miR-1-3p; hsa-miR-503-5p
hsa-miR-1-3p; hsa-miR-191-5p; hsa-miR-200b-3p; hsa-miR-503-5p
hsa-miR-31-3p; hsa-miR-1-3p; hsa-miR-191-5p
hsa-miR-1-3p; hsa-miR-191-5p
hsa-miR-1-3p; hsa-miR-191-5p
hsa-miR-31-3p; hsa-miR-1-3p; hsa-miR-191-5p; hsa-miR-200b-3p; hsa-miR-503-5p
hsa-miR-1-3p; hsa-miR-191-5p; hsa-miR-200b-3p; hsa-miR-503-5p
hsa-miR-1-3p; hsa-miR-191-5p
hsa-miR-31-3p; hsa-miR-1-3p; hsa-miR-191-5p; hsa-miR-200b-3p; hsa-miR-503-5p
hsa-miR-1-3p; hsa-miR-200b-3p; hsa-miR-503-5p
hsa-miR-31-3p; hsa-miR-1-3p; hsa-miR-191-5p; hsa-miR-200b-3p; hsa-miR-503-5p
hsa-miR-31-3p; hsa-miR-1-3p; hsa-miR-191-5p; hsa-miR-200b-3p; hsa-miR-503-5p
hsa-miR-1-3p; hsa-miR-191-5p; hsa-miR-200b-3p; hsa-miR-503-5p
