# Supplementary material for: miRNA Expression Profiles in Ovarian Endometriosis and Two Types of Ovarian Cancer—Endometriosis-Associated Ovarian Cancer and High-Grade Ovarian Cancer
Source: Int J Mol Sci. 2023 Dec 14;24(24):17470. doi: 10.3390/ijms242417470 (PMC10743418; doi:10.3390/ijms242417470)
Supplement: Supplementary file 1 [file ijms-24-17470-s001.zip › Table S1.pdf]

miRNA \ Groups	Control group	Ovarian endometrial cyst	EOC	HGSOC
miR-1-3p<br>Assay ID: 477820_mir	1	1.4029	1.0037	1.0615
miR-31-3p<br>Assay ID: 478012_mir	1	0.9693	8.6679	0.9646
miR-125b-1-3p<br>Assay ID: 478665_mir	1	1.2257	0.1475	0.2446
miR-200b-3p<br>Assay ID: 477963_mir	1	0.2539	15.0699	7.9167
miR-548d<br>Assay ID: 480870_mir	1	1.5972	2.1021	0.8198
miR-502-5p<br>Assay ID: 47954_mir	1	0.1191	0.0055	0.1477
miR-503-5p<br>Assay ID: 47143_mir	1	0.005	0.1759	0.5978

Table S1. Names, Assays IDs, and Relative expression levels of the miRNAs studied on pooled groups of ovarian endometriosis, EOC, HGSOC, healthy subjects. n=20 for each group; values shown as RQ.
